# Diagnostic Criteria for Obesity Disease in Cats

**DOI:** 10.3389/fvets.2019.00284

**Published:** 2019-08-27

**Authors:** Yuki Okada, Hiromichi Ueno, Takayuki Mizorogi, Kenji Ohara, Koh Kawasumi, Toshiro Arai

**Affiliations:** ^1^School of Veterinary Medicine, Nippon Veterinary and Life Science University, Musashino, Japan; ^2^Japan Animal Medical Center, Shibuya, Japan

**Keywords:** adiponectin, cat, metabolically healthy obesity, obesity disease, SAA

## Abstract

Accumulated visceral and subcutaneous fat masses were measured with computed tomography (CT) in cats with various body condition scores (BCS) from 5/9 to 9/9. BCS does not always reflect visceral fat accumulation which induces pro-inflammatory reactions. Obese cats with accumulated visceral fat showed low plasma adiponectin and high serum amyloid A (SAA) concentrations, an inflammatory marker. Based on the above results, new diagnostic criteria for obesity disease were established as follows. For overweight cats with high BCS of >7/9, showing two or more of the following three symptoms, low adiponectin concentrations, hyperlipidemia, and high SAA concentrations, categorizes them as having obesity disease. Cats with BCS 6/9–9/9, without inflammatory reactions, were classified as simple obesity, which is similar to metabolically healthy obesity (MHO) defined in human medicine. Simple obesity group showed significantly higher adiponectin concentrations than those in control group. The obesity disease group showed significantly higher plasma triglyceride (TG) and SAA concentrations and lower concentrations of adiponectin than the control group. Moreover, plasma glucose and malondialdehyde (MDA) concentrations in the obesity disease group were higher than those in healthy control group, although the differences were not statistically significant. Establishing criteria for obesity disease based on visceral fat accumulation and inflammation markers levels contributes to early and correct diagnosis of obesity in cats.

## Introduction

Obesity is a serious public health concern all over the world. According to a report form World Health Organization (WHO), it was estimated that over 1.9 billion human adults (39%) worldwide were overweight, of which more than 650 million (~13%), were obese in 2016 (1). Life spans of dogs and cats have been prolonged for past decades owing to the improvement of veterinary medicine and husbandry. Prevalence of obesity in dogs and cats has increased accompanying with aging in these years as in humans (2, 3). Cats are prone to become obese compared to dogs owing to their unique characteristics in glucose and lipid metabolism (4, 5). Prevalence of obesity in cats is assumed to be 30–40% (3, 6). Obesity is classified into two types, without and with health issues (7). Obesity accompanied by health issues is defined as obesity diseases (pathological obesity) in human medicine (8, 9). Visceral fat accumulation causes insulin resistance and is a risk factor for metabolic syndrome, diabetes mellitus, hypertension, dyslipidemia, and some types of cancer (10, 11), and overweight and obesity are usually associated with shorter lifespan (12, 13). Visceral fat is considered to be metabolically active compared to subcutaneous fat, and releases various pro-inflammatory cytokines and fatty acids (14, 15). Increased visceral fat contributes to increased incidence of metabolic syndrome (16, 17). Increased waist circumference, reflecting mass of visceral fat, is one criterion for metabolic syndrome and obesity disease in human medicine (18, 19). Many studies to date have shown high levels of acute phase proteins, high-sensitive C-reactive protein (hs-CRP), to reflect inflammation, among patients with metabolic syndrome (20). In cats, serum amyloid A (SAA) is considered to be a sensitive biomarker for inflammatory reactions (21).

Meanwhile, a subgroup of individuals with obesity does not seem to be at an increased risk for metabolic complications of obesity, and the obesity sub-phenotypes are referred as metabolically healthy obesity (MHO) in human medicine (22–24). Obese cats without significant health issue are sometime observed, and those cats might be categorized to MHO.

In this study, we measured changes in plasma concentrations of biomarkers (metabolites, inflammatory markers, hormones, and enzymes) and visceral fat mass using computed tomography (CT) in cats with various body condition scores (BCS). The aim of this study is to define obesity disease in cats using CT images and plasma biomarkers to diagnose obesity correctly at its early stage.

## Materials and Methods

### Animals

Ten client-owned cats (2–12 years old, castrated male, eight mix breeds, one American shorthair, one Russian Blue) with varying BCS from a veterinary hospital in Tokyo were entered into the study. Body weight and body condition score (on scale from 1 to 9 where 1 is emaciated, 5 is ideal, and 9 is extremely fat) (25) were measured. Informed consent was obtained from each client in the written form. The informed consent included information about the possible risk, benefits, and limits of examination. Ten healthy control cats (six female, four male, 2–3 years old, mix breeds) were maintained for laboratory experiments at Narita Animal Science Laboratory Co., Ltd. (Narita, Chiba) and fed commercial diets. Ethical approval for this study was from Narita Animal Science Laboratory Co., Ltd., Research Animal Ethical Committee (19–05).

### Blood Sampling and Metabolite, Hormone, and Enzyme Assay

Preprandial blood was collected from jugular vein, and plasma and serum were separated by centrifugation with and without heparin as anti-coagulant. Plasma and serum samples were stored at −80°C until use. Plasma glucose, triglyceride (TG) concentrations and aspartate aminotransferase (AST) and alanine aminotransferase (ALT) activities were measured using autoanalyzer (JCA-BM2250, JEOL Ltd., Tokyo, Japan) with the manufacture's reagents at FUJIFILM Monolith Co., Ltd. (Tokyo, Japan). Plasma non-esterified fatty acids (NEFA) concentrations were measured with a commercial kit (NEFA-C Test, Wako Pure Chemical Industries, Ltd., Tokyo, Japan). Plasma malondialdehyde (MDA) and adiponectin concentrations were measured with a commercial kit, NWLSSTM Malondialdehyde assay kit (Northwest Life Science Specialties, LLC, Vancouver, Canada) and mouse/rat adiponectin ELISA kit (Otsuka Pharmaceutical Co., Ltd., Tokyo, Japan), respectively. Serum amyloid A (SAA) concentrations were measured with a commercial kit, CAT SERUM AMYLOID A (SAA) ELISA (Life Diagnostics, Inc., West Chester, PA, USA).

### Measurement of Subcutaneous and Visceral Fat Areas by CT

The subcutaneous and visceral fat areas (SFA and VFA) were measured using cross-sectional computed tomography (CT) images. Client-owned feline subjects (two each from BCS 5, 6, 7, 8, and 9/9 groups) were retained in ventral recumbency without anesthesia. The CT images were obtained at three locations, 3rd, 5th, and 7th lumber vertebra (L3, L5, and L7) of the subjects using a multi-slice CT (BrightSpeed eight rows, GE Healthcare, Tokyo, Japan). Imaging conditions were as follows; a tube voltage of 120 kV, tube current of 118 mA, and imaging slice thickness of 1.25 mm. Scanned images were reconstructed by RETRO reconstruction function of the CT scanner, and processed using a built-in scanner. The images were uploaded to a separate workstation on which the OsiriX software was installed. The subcutaneous fat area (SFA, cm^2^) and visceral fat area (VFA, cm^2^) were calculated using the OsiriX software.

### Statistics

Measured values are expressed as means ± standard error (SE). Statistical significance was determined by paired-*t*-test. The significance level was set at *p* < 0.05.

## Results

**Figure 1:**
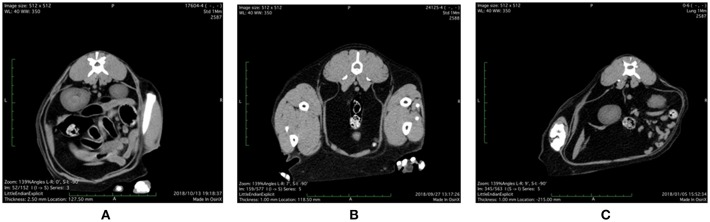
Abdominal CT images of **(A)** control, **(B)** simple obesity, and **(C)** obesity disease cats at L3 positions. **(A)** Control, BCS 5/9; VFA 4.3 cm^2^, SFA 19.8 cm^2^, V/SA = 0.22; Adiponectin 3.8 μg/mL; **(B)** Simple obesity, BCS 9/9, VFA 26.6 cm^2^, SFA 43.6 cm^2^, V/S = 0.60; Adiponectin 21.9 μg/mL; **(C)** Obesity disease, BCS 7/9, VFA 57.5 cm^2^, SFA 24.7 cm^2^, V/S = 2.33; Adiponectin 2.0 μg/mL. VFA, visceral fat area; SFA, subcutaneous fat area; V/S, VFA/SFA ratio.

Representative abdominal CT images of cats with various health and metabolic status are shown in Figure 1. In the healthy control cat with BCS 5/9 and plasma adiponectin concentration, 3.8 μg/mL of visceral fat area (VFA, 4.3 cm^2^) was less than subcutaneous fat area (SFA, 19.8 cm^2^) and VFA/SFA (V/S) ratio was 0.22. The simple obesity cat with BCS 9/9 had high plasma adiponectin concentrations (21.9 μg/mL), and larger VFA (26.6 cm^2^) and SFA (43.6 cm^2^) than the healthy control. The V/S ratio was 0.60, which was slightly higher than that of the healthy control. The obesity disease cat with BCS 7/9 showed larger VFA (57.5 cm^2^) than SFA (24.7 cm^2^), V/S ratio of 2.33, and the plasma adiponectin concentrations under 3.0 μg/mL.

From the above abdominal CT images and metabolites and hormones concentrations, we present the diagnostic criteria for obesity disease as shown in Table 1. Obesity disease may be diagnosed if overweight cats with BCS >7/9 show two or more of the following, low adiponectin (<3 μg/mL), hyperlipidemia (TG > 165 mg/100 mL) and high SAA values (>200 ng/mL). According to this criteria, out of 10 cats studied with CT, four cats with BCS 6/9–9/9 were diagnosed as obesity diseases, while another four cats with BCS 6/9–9/9 were classified to simple obesity.

**Table 1:** Diagnostic criteria of obesity disease in cats.

Overweight (BCS > 7/9)
with two or more of the following symptoms
- Low adiponectin (<3 μg/mL)
- Hyperlipidemia (TG > 165 mg/100 mL)
- High SAA (>200 ng/mL)

Comparison of metabolites and hormone concentrations, enzyme activities in cats are shown in Table 2. Simple obesity group showed significantly higher adiponectin concentrations than those in control group. Plasma glucose, TG, NEFA concentrations, and AST and ALT activities were similar to those in control. The obesity disease group showed significantly higher plasma TG and SAA concentrations and lower concentrations of adiponectin than the control group. Moreover, plasma glucose and MDA concentrations in the obesity disease group were higher than those in the health control group, however the difference was not statistically significant.

**Table 2:** Comparison of fasting metabolites and hormone concentrations and enzyme activities in plasma of cats.

	**Control (*n* = 10)**	**Simple obesity (*n* = 4)**	**Obesity disease (*n* = 4)**
BCS	5.1 ± 0.2	7.8 ± 0.8^*^	7.7 ± 0.3^*^
BW (kg)	3.4 ± 0.2	7.1 ± 1.0^*^	6.2 ± 0.4^*^
Glucose (mg/100 mL)	84.0 ± 3.0	95.0 ± 10.6	120.3 ± 2.9
Triglyceride (mg/100 mL)	38.5 ± 2.0	40.0 ± 3.3	385.7 ± 308.1^*^
NEFA (mEq/L)	0.73 ± 0.09	0.47 ± 0.09	0.52 ± 0.11
MDA (μmol/L)	1.89 ± 0.15	1.35 ± 0.17	2.47 ± 0.84
SAA (ng/mL)	29.2 ± 8.2	14.5 ±14.5	469.7 ± 448.3
Adiponectin (μg/mL)	5.6 ± 1.0	15.0 ± 3.2^*^	3.2 ±0.8^*^
AST (IU/L)	27.5 ± 1.3	24.8 ± 4.7	27.3 ± 9.2
ALT (IU/L)	42.4 ± 3.7	35.5 ± 2.1	37.7 ± 10.7

*Values are presented as means ± SE*.

* *Significantly different (p <0.05) from Control group*.

## Discussion

Obesity is defined as excessive accumulation of triglyceride (TG) in adipose tissue, due to an energy imbalance where energy intake exceeds energy expenditure. However, there are varying types of obesity in animals. In this study, we proved that being overweight is not always pathological in cats. Body condition scores reflect the degree of fat accumulation in the whole body, but not the distribution of accumulated fat in visceral tissues. Accumulation of visceral fat can induce lipotoxicity (26). Visceral fat is more active than subcutaneous fat and releases substances, including harmful adipokines such as tumor necrosis factor α (TNF-α), interleukin-6, and others (27, 28), and visceral fat accumulation is linked to inflammation. It is important to evaluated increasing visceral fat to understand the etiology of obesity correctly.

In this study, feline subjects were retained in face-down position without anesthesia at CT, therefore images contain not only abdomen but extra tissues such as skeletal muscles. In human medicine, a single slice at the umbilical level is usually used for CT examination of abdominal fat (29). In this study, CT images at L3 was used for measuring the subcutaneous and visceral adipose tissue areas. CT images at L3 showed subcutaneous and visceral adipose tissues in abdomen more clearly compared to those obtained at L5 and L7. Further studies are necessarily to determine the best CT study points appropriate for CT examination of abdominal fat in more species.

Serum amyloid A (SAA), major feline acute-phase protein (APP), is reported to increase during early stage inflammation and with tissue damage (30). Inflammation associated with obesity is very small and increase in SAA values is considered to be minor. The measuring kit for SAA used in this study is very sensitive compared to the previous measuring systems (31), so a slight increase in SAA accompanying obesity disease seemed to be detected. In obesity disease cats, plasma adiponectin concentrations decreased significantly and plasma TG concentrations increased significantly, and SAA concentrations were variable among the subjects, from 200 to over 1,000 ng/mL. The cat with over 1,000 ng/mL of SAA, stomatitis was found. Significantly high SAA concentrations in this study were possibly caused by other inflammatory process rather than obesity.

Some of cats with high BCS > 7/9 were found to have high plasma adiponectin concentrations and normal levels of TG and liver enzyme activities, and those cats showed low SAA values. In human medicine, obese individual without the common obesity-associated metabolic abnormalities, such as dyslipidemia, insulin resistance, hypertension, and unfavorable inflammatory profile (32, 33) are classified as metabolically healthy obesity (MHO) (24). From these viewpoints, the above simple obesity may be classified as metabolically healthy obesity. MHO can quickly progress to unhealthy obesity (22, 24), and reduction of body weight is advised for maintenance of their healthy condition. To categorize MHO in cats, further studies are needed in more obese cats.

Obesity results in increased inflammation and oxidative stress which cause genomic damages and mitochondrial dysregulation (34, 35). Lipid peroxidation by reactive oxygen species (ROS) is accelerated in obese animals (36). Cats with obesity disease showed higher concentration of plasma MDA, a lipid peroxidation marker, than control and MHO cats. It has been reported that supplementation of anti-oxidant compounds are effective for improvement of lipid metabolism in obese dogs (37). As the treatment of obesity disease, anti-oxidant compounds supplement is considered to be useful also in cats.

This is a preliminary study to classify obesity in cats. Limitations of our study include small number of samples and biological and environmental variables (age, sex, diet), which are inevitable when client-owned animals from one veterinary hospital are utilized. As castration is one of factors contributing to the development of obesity in male cats (38, 39), further study is necessary with more animals with various age and sex in various districts to evaluate utility of the criteria of obesity diseases. Also, in this study, ADN antibody used for measurement was not specific to the feline species, and there is no established reference values for feline ADN, which is not measured using the specific antibody for feline AND. Therefore, the diagnostic criteria shown in Table 1 are considered provisional.

In cats, prevalence of obesity has increased dramatically in the past decades, making obesity a major public concern. To reduce the detrimental effects of obesity and its associated diseases, early and correct identification of at-risk individuals is imperative. Establishing criteria for obesity disease based on visceral adipose tissue accumulation and levels of inflammatory markers will help correctly diagnose obesity in cats.

## Conclusion

Accumulated visceral and subcutaneous fat amounts were measured with computed tomography (CT) in cats with various body condition score (BCS) from 5/9 to 9/9. BCS does not always reflect accumulated area of visceral fat which induce pro-inflammatory reactions. Obese cats with accumulated visceral fat showed low plasma adiponectin concentrations and high concentrations of SAA, an inflammatory marker. Based on the above results, new diagnostic criteria for obesity disease are made as the following. In cats with high BCS of >7/9, showing two or more of the following three symptoms; low adiponectin concentrations, hyperlipidemia and high SAA concentrations, will classified them as obesity disease. Establishing criteria for obesity disease based on the amount of visceral fat and levels of inflammation markers will help correctly diagnose obesity in cats.

## Data Availability

All datasets generated for this study are included in the manuscript/supplementary files.

## Author Contributions

YO and HU contributed to the conception and design of the work and drafted the work. TM, KO, and KK contributed to the acquisition, analysis, and interpretation of data. TA contributed to the design of the work and final approval of the version to be publication. All authors read and approved the final work.

### Conflict of Interest Statement

The authors declare that the research was conducted in the absence of any commercial or financial relationships that could be construed as a potential conflict of interest.

## Abbreviations

ALT: alanine aminotransferase
AST: aspartate aminotransferase
CT: computed tomography
MDA: malondialdehyde
MHO: metabolically healthy obesity
NEFA: non-esterified fatty acid
SFA: subcutaneous fat area
SAA: serum amyloid A
TG: triglyceride
VFA: visceral fat area.
